# Post-Synthesis Heat Treatment Induces Polymorphic Homogenization and Band-Tail Narrowing in Sol–Gel TiO_2_ Nanostructures

**DOI:** 10.3390/nano16211369

**Published:** 2026-10-27

**Authors:** Angiee C. Jiménez-Oñate, Ana B. González-Rojas, Edgar D. Peduzine-Orozco, Juan P. Molina-Jiménez, Duber A. Avila, Sindi D. Horta-Piñeres

**Affiliations:** Grupo de Óptica e Informática, Universidad Popular del Cesar, Valledupar 200001, Colombia; angieecjimenez@unicesar.edu.co (A.C.J.-O.); anabgonzalez@unicesar.edu.co (A.B.G.-R.); epeduzine@unicesar.edu.co (E.D.P.-O.); duberavilap@unicesar.edu.co (D.A.A.); shorta@unicesar.edu.co (S.D.H.-P.)

**Keywords:** TiO_2_ nanostructures, sol–gel synthesis, heat treatment, anatase, brookite, optical band gap, Urbach energy, Rietveld

## Abstract

Post-synthesis heat treatment provides a route for tuning the structural and optical response of TiO_2_ without necessarily altering its particle architecture. In this study, TiO_2_ nanostructures prepared by the sol–gel method were evaluated before and after a stepwise heat treatment from 250 to 400 °C using XRD/Rietveld, Raman spectroscopy, FE-SEM/EDS, statistical morphometry, and UV–Vis diffuse reflectance spectroscopy, with XPS performed on TiO_2_-400. TiO_2_-250 consisted of 96.76 ± 1.22 wt.% anatase and 3.24 ± 0.47 wt.% brookite, whereas anatase was the only crystalline phase detected in TiO_2_-400 within the XRD detection limit. Concurrently, the coherent crystalline domain size increased from 44.3 ± 2.6 to 53.6 ± 1.9 nm, without a statistically significant increase in the equivalent circular diameter of the particles. The apparent optical band gap increased from 3.14 ± 0.03 to 3.25 ± 0.03 eV, while the Urbach energy decreased by approximately 48%, from 189.4 ± 1.1 to 98.0 ± 0.4 meV. These results show that moderate heat treatment is accompanied by polymorphic homogenization and predominantly intraparticle crystalline/electronic reorganization, together with narrowing of the band-tail distribution without appreciable external particle growth. Overall, the study establishes a multiscale correlation between phase evolution, coherent-domain growth, morphological stability, and optical-edge narrowing in sol–gel TiO_2_ nanostructures.

## 1. Introduction

Titanium dioxide (TiO_2_) is a semiconductor oxide whose physicochemical properties are strongly determined by its crystal structure, characteristic dimensions, surface chemistry, and the distribution of electronic states associated with defects. Its main polymorphs, anatase, brookite, and rutile, exhibit different arrangements and degrees of distortion of the TiO_6_ octahedra, resulting in distinct crystallographic symmetries, energy stabilities, and electronic structures [1]. These differences influence the optical response, charge carrier dynamics, and surface reactivity of TiO_2_, properties that are relevant for applications such as photocatalysis, environmental remediation, energy conversion, and sensors. Furthermore, in multiphase systems, interactions between polymorphs and their interfacial regions can alter the effective response compared to the phases considered in isolation.

Among the various synthesis routes, the sol–gel method allows for control of the hydrolysis, condensation, nucleation, and subsequent crystallization processes of TiO_2_ under relatively mild conditions. Titanium (IV) isopropoxide (TTIP) is one of the most widely used precursors, and its transformation into Ti–O–Ti lattices depends on variables such as the water-to-alkoxide ratio, the solvent, the pH, the temperature, and the presence of complexing agents or acidic catalysts [2]. These conditions affect particle formation, phase composition, and the degree of structural order achieved after heat treatment. In particular, small variations in the sol chemistry can lead to single-phase anatase or anatase/brookite mixtures [3,4], so the presence of a minor amount of brookite may directly reflect the hydrolysis, nucleation, and growth conditions established during synthesis.

Post-synthesis heat treatment introduces an additional level of structural control by promoting the condensation of residual Ti–OH groups, the removal of precursor-derived species, the relaxation of lattice distortions, the growth of coherently diffracting domains, and the redistribution of metastable polymorphs. Thermally induced changes in the anatase/brookite ratio and in their optical and photocatalytic properties have been reported [5,6]; however, most of these studies have focused on optimizing the phase ratio, establishing transformation temperatures, or evaluating photocatalytic performance. Comparatively less studied is how a minor fraction of brookite evolves within a material that is already predominantly anatase under moderate heat treatments and, particularly, whether such polymorphic homogenization is accompanied by external particle growth or can occur primarily through the reorganization of crystalline domains within an essentially preserved morphological envelope.

This distinction is also relevant for interpreting the optical response, since there is no universal relationship between temperature, crystal growth, and band gap energy (*E_g_*). In sol–gel-synthesized TiO_2_, decreases, increases, or insignificant variations in *E_g_* have been reported, depending on the phase composition, the degree of disorder, and the population of defect states [7]. Therefore, the increase in crystallite size alone does not sufficiently explain the evolution of the optical edge. In this context, the Urbach energy (*E_U_*) provides complementary information on the energy width of the exponential tail of absorption associated with localized states near the band edge. In nanoscale TiO_2_, higher degrees of structural or electronic disorder have been linked to higher *E_U_* values and lower apparent optical band gap energies, particularly when pre-edge absorption contributes significantly to the optical response [8,9]. Consequently, thermal evolution must be analyzed by jointly considering phase composition, crystalline domains, external morphology, and tail electronic states.

Despite the extensive structural and optical characterization of thermally treated TiO_2_, the quantitative relationship between polymorphic homogenization, crystalline domain growth, morphological stability at the particle scale, and the evolution of localized optical states during moderate post-synthesis treatments of already crystalline materials rich in anatase remains less well established. This study investigates this relationship in TiO_2_ nanostructures obtained via the sol–gel method, using a portion of the material initially treated at 250 °C as a direct precursor for a subsequent stepwise treatment up to 400 °C. Rietveld refinement and Halder–Wagner analysis were combined with Raman spectroscopy, XPS, FE-SEM/EDS, statistical morphometry, and diffuse reflectance UV–Vis spectroscopy. The results show that the evolution from an anatase-rich system with a minor amount of brookite toward a state in which anatase is the only crystalline phase detected by XRD occurs in conjunction with the growth of crystalline domains, but without a statistically significant increase in the external particle size, and is accompanied by a marked decrease in *E_U_* and by the evolution of the optical edge toward the response characteristic of anatase. Taken together, these results indicate a predominantly intraparticle reorganization that occurs concurrently with polymorphic homogenization and a quantifiable narrowing of the distribution of localized electronic states near the optical edge.

## 2. Materials and Methods

### 2.1. Synthesis of TiO_2_ Nanostructures

**Figure 1:**
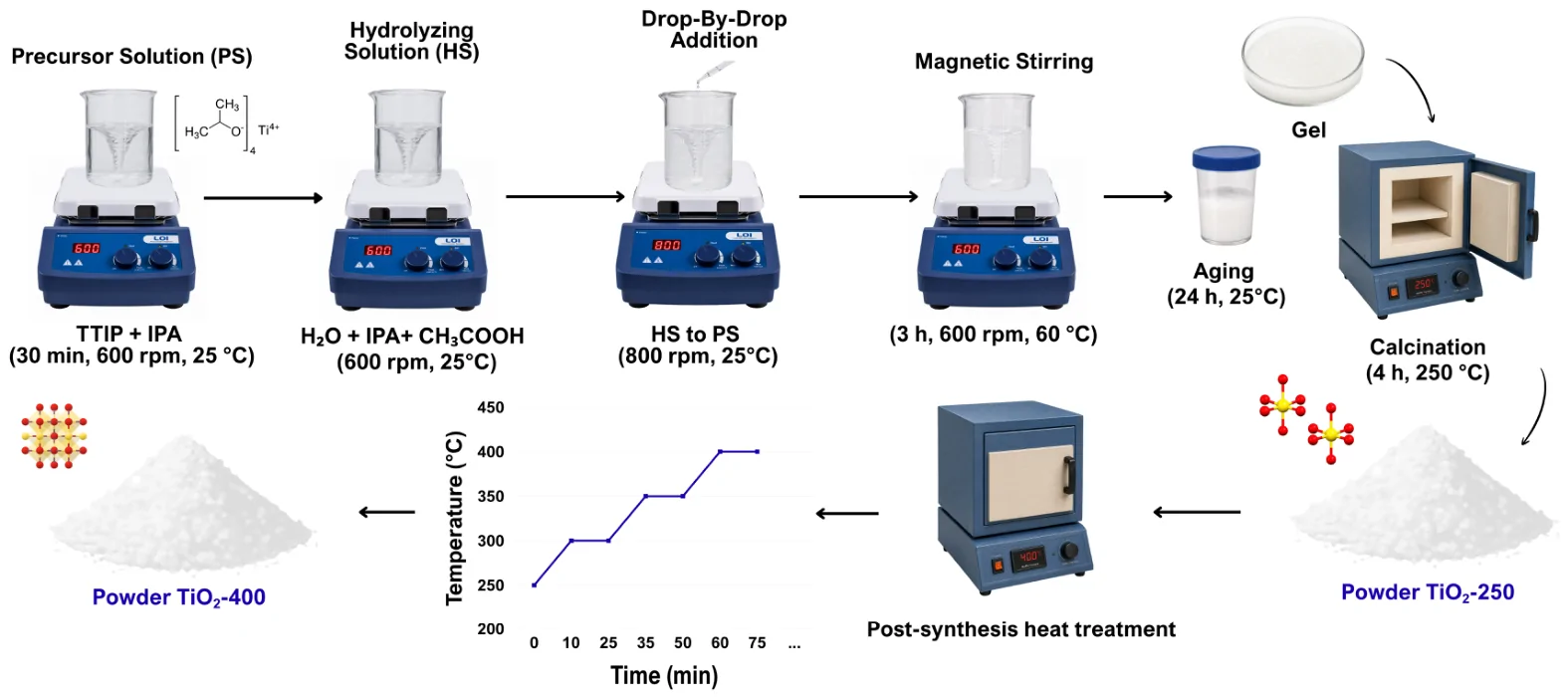
Schematic illustration of the sol–gel synthesis and post-synthesis heat-treatment route used to obtain TiO_2_-250 and TiO_2_-400 nanostructures. The post-synthesis thermal sequence indicates the setpoints and holding periods; transition times between setpoints are not represented because the heating rate was not recorded.

TiO_2_ nanostructures were synthesized by the sol–gel method following the procedure outlined in Figure 1. Titanium (IV) isopropoxide (TTIP, 97%, Sigma-Aldrich, Merck KGaA, Darmstadt, Germany) was used as the titanium precursor; isopropyl alcohol (IPA, 99.8%, Merck KGaA, Darmstadt, Germany) was used as the solvent, while glacial acetic acid (CH_3_COOH, ≥99.8%, Merck KGaA, Darmstadt, Germany) served as the acid catalyst and complexing agent during the sol–gel process. Distilled water was used as the hydrolyzing agent.

First, a precursor solution (PS) was prepared by dissolving 10 mL of TTIP in 40 mL of isopropyl alcohol, keeping the mixture under continuous magnetic stirring at 600 rpm for 30 min at room temperature (25 ± 2 °C), until a homogeneous solution was obtained. Separately, a hydrolyzing solution (HS) was prepared by mixing 5 mL of distilled water with 20 mL of isopropyl alcohol, followed by the addition of 2 mL of glacial acetic acid. The HS solution was slowly added to the precursor solution drop by drop (≈1 drop s^−1^) under magnetic stirring at 800 rpm. Once the addition was complete, the mixture was kept under continuous stirring at 600 rpm for 3 h at 60 ± 2 °C to promote the hydrolysis and condensation reactions of the titanium precursor. Subsequently, the system was allowed to age for 24 h in a hermetically sealed vessel.

The resulting gel was transferred to a muffle furnace and heat-treated in air at 250 °C for 4 h. After this process, the sample was allowed to cool naturally inside the muffle furnace until it reached room temperature, and the resulting solid was carefully ground in an agate mortar until a homogeneous powder was obtained, designated TiO_2_-250.

To evaluate the effect of post-synthesis heat treatment, a portion of the TiO_2_-250 powder was subjected to a stepwise thermal treatment in a Thermo Scientific Thermolyne F6010 muffle furnace (Thermo Fisher Scientific, Waltham, MA, USA). The sample was held for 15 min at 250, 300, and 350 °C and for 2 h at 400 °C. After each hold, the setpoint was manually increased, and the next hold began once the new temperature was reached. No fixed heating rate was programmed, and the transient heating rate was not recorded. The sample then cooled naturally inside the furnace to room temperature and was designated TiO_2_-400. The complete process is schematically presented in Figure 1.

### 2.2. Characterization of TiO_2_ Nanostructures

To conduct a comprehensive study of the structural evolution of TiO_2_ nanostructures synthesized via the sol–gel method following heat treatment, their structural, vibrational, compositional, morphological, and optical properties were investigated.

#### 2.2.1. X-Ray Diffraction Measurements and Structural Refinement

The crystallinity and structural parameters of the TiO_2_-250 and TiO_2_-400 nanostructures were determined by X-ray diffraction (XRD) using a Malvern PANalytical Empyrean 2012 X-ray diffractometer (Malvern Panalytical, Malvern, UK), equipped with Cu Kα_1_ radiation (λ = 1.541 Å), a PIXcel3D detector, and configured in reflection–transmission mode with a spinner system. The diffractograms were recorded at room temperature using a voltage of 45 kV, a current of 40 mA, an angular step of 0.05°, and a counting time of 50 s per step.

Structural refinement was performed using the Rietveld method with the FullProf Suite software (version 5.20) [10]. The following parameters were refined: the scale factor, the background, the angular zero shift, the lattice parameters, the Caglioti *U*, *V*, and *W* profile parameters, the atomic positions, the isotropic displacement factors (B_iso_), and the occupation factors.

The quality of the refinements was evaluated using the reliability factors *R_p_*, *R_wp_*, *R_exp_* and χ^2^. To examine possible changes in the preferred crystallographic orientation, the Harris texture coefficient (*η_hkl_*) was calculated for the reflections (101), (004), (200), (105), (211), and (204), using the following equation [11]:

(1) $$\eta_{\mathrm{hkl}}=\frac{I_{\mathrm{hkl}}/I_{\left(\mathrm{hkl}\right)}^{0}}{\frac{1}{N}\sum_{i=1}^{N}I_{\mathrm{hkl}}/I_{\left(\mathrm{hkl}\right)}^{0}}$$

where *I_hkl_* and $I_{\left(\mathrm{hkl}\right)}^{0}$ correspond to the experimental and standard (ICDD) intensities, respectively, and *N* corresponds to the number of reflections considered.

Microstructural evolution was evaluated using the Halder–Wagner method [12], which is used to simultaneously estimate the average size of coherent crystalline domains (*D*) and lattice microstrain (*ε*) based on the broadening of diffraction peaks. The analysis was performed using the following equation:

(2) $${\left(\frac{\beta_{\mathrm{hkl}}^{*}}{d_{\mathrm{hkl}}^{*}}\right)}^{2}=\frac{1}{D_{\mathrm{HW}}}\frac{\beta_{\mathrm{hkl}}^{*}}{{d_{\mathrm{hkl}}^{*}}^{2}}+\frac{\varepsilon^{2}}{4}$$

where

$$\beta_{\mathrm{hkl}}^{*}=\frac{\beta \cos(\theta )}{\lambda },\;d_{\mathrm{hkl}}^{*}=\frac{2\sin(\theta )}{\lambda }$$

where *β* is the integral reflection broadening corrected for instrumental broadening, expressed in radians; *θ* is the Bragg angle; and *λ* is the wavelength of the incident radiation. Plotting ${\left(\beta^{*}/d^{*}\right)}^{2}$ versus $\beta^{*}/{\left(d^{*}\right)}^{2}$ allowed us to determine *D* from the inverse of the slope and *ε* from the y-intercept. Complementary to this, three-dimensional models of the crystal structures and selected planes were constructed using the refined structural parameters and visualized using the VESTA software (version 3.5.7) [13].

#### 2.2.2. Raman Spectroscopy Measurements

The vibrational properties of the TiO_2_-250 and TiO_2_-400 nanostructures were examined using an Rm1 Raman spectrometer (ASEQ Instruments, Vancouver, BC, Canada), equipped with a 532 nm excitation laser with a power of 5.0 mW, a spectral operating range of 200–3100 cm^−1^, and a resolution of 4 cm^−1^. Three independent measurements were performed for each sample under the same experimental conditions, and the comparative analysis focused on the 300–750 cm^−1^ region, where the characteristic vibrational modes of the anatase phase were identified. For each replicate, band fitting was performed using Voigt profile functions to determine the spectral position and full width at half maximum (FWHM) of the identified bands.

#### 2.2.3. X-Ray Photoelectron Spectroscopy (XPS) Measurements

The surface chemical state of TiO_2_-400 was investigated using X-ray photoelectron spectroscopy (XPS); no XPS measurements were acquired for TiO_2_-250. A SPECS spectrometer (SPECS Group, Berlin, Germany) was used for the measurements; it is equipped with a PHOIBOS 150 1D-DLD analyzer and a monochromatic Al Kα (1486.7 eV) source. Survey and high-resolution (HR) spectra were acquired using step energies of 85 and 20 eV, respectively. The step size was 1 eV for the survey spectra and 0.1 eV for the high-resolution spectra. Five acquisition cycles were performed for the survey spectra and twenty cycles for the high-resolution spectra. Charge compensation of 3 eV and 20 mA was used. The binding energy scale was referenced using the C 1s signal from adventitious carbon.

#### 2.2.4. FE-SEM, EDS, and Morphometric Characterization

The morphological characteristics and elemental composition of the TiO_2_-250 and TiO_2_-400 nanostructures were analyzed by field-emission scanning electron microscopy (FE-SEM), using a Scios 2 LoVac dual-beam FIB–FESEM microscope (Thermo Fisher Scientific, Waltham, MA, USA). The instrument is equipped with an X-ray energy-dispersive spectroscopy (EDS) microanalysis system (UltraDry, 129 eV resolution, 30 mm^2^ detector; model SDBX-30PM-B). The micrographs used for morphological characterization were acquired at an acceleration voltage of 5 kV. To compare the two samples, representative images acquired at 30,000× magnification were used, supplemented by micrographs at a higher magnification of 100,000× to visualize the contours and organization of the particles in greater detail.

The morphometric analysis was performed on FE-SEM micrographs using equivalent selection criteria for TiO_2_-250 and TiO_2_-400, excluding overlapping, coalesced, truncated, or ambiguously shaped particles. For each particle, the equivalent circular diameter ($\mathrm{ECD}=2\sqrt{A/\pi }$), circularity (4 *πA*/*P*^2^), aspect ratio (AR), and solidity were determined. The distributions were represented using histograms and kernel density estimates (KDE); after assessing their normality, the differences between the two samples were analyzed using the nonparametric Mann–Whitney U test. The EDS analysis was performed under the same nominal conditions for both samples, using 10 kV, an approximate magnification of 59,942×, and a resolution of 512 × 340 pixels (≈0.01 µm pixel^−1^). Ti and O were quantified as weight and atomic percentages normalized to these two elements. Carbon was not included in the EDS quantitative normalization. Because oxygen quantification by EDS is semiquantitative, the derived O/Ti ratios were used only as descriptive comparative parameters and not to infer absolute stoichiometry or oxygen-vacancy concentrations.

#### 2.2.5. UV–Vis Diffuse Reflectance Spectroscopy Measurements

The optical properties of TiO_2_-250 and TiO_2_-400 were evaluated by UV–Vis diffuse reflectance spectroscopy using a fiber-optic UV–Vis spectrometer (ASEQ Instruments, Vancouver, BC, Canada), with a spectral resolution of 0.2 nm, an operating range of 200–1000 nm, and an F01_R03 reflection probe.

The Kubelka–Munk function was calculated from diffuse reflectance measurements, following the methodological considerations described for particulate semiconductors [14,15]:

(3) $$F(R_{\infty })=\frac{K}{S}=\frac{{\left(1-R_{\infty }\right)}^{2}}{2R_{\infty }}$$

where *K* and *S* represent the absorption and scattering coefficients, respectively, and *R_∞_* corresponds to the relative reflectance of an optically thick sample. Given the characteristic indirect optical transition of TiO_2_ anatase, the apparent optical band gap energy (*E_g_*) was determined from the Tauc plot ${\left[F\left(R_{\infty }\right)hv\right]}^{1/2}$ versus *hν*. Due to the presence of a pre-edge contribution, the value of *E_g_* was obtained from the intersection between the linear fit of that region and the linear fit corresponding to the Tauc edge [16].

The absorption tail near the optical edge was analyzed according to the exponential behavior described by Urbach [17]. For materials analyzed using diffuse reflectance, and under the assumption of a weakly energy-dependent scattering contribution in the region under consideration, the Kubelka–Munk function can be used as a quantity proportional to absorption. Consequently, the Urbach energy was determined using:

(4) $$\ln[F(R_{\infty })]=\ln(F_{0})+\frac{\mathrm{hv}}{E_{U}}$$

where *E_U_* corresponds to the energy width of the exponential tail and was obtained as the inverse of the slope of the linear fit of ln[*F*(*R_∞_*)] versus *hν*.

The uncertainties in *E_g_* were obtained directly from the fit, and those in *E_U_* were obtained by propagating the error associated with the slope.

## 3. Results and Discussion

### 3.1. Structural Analysis

**Figure 2:**
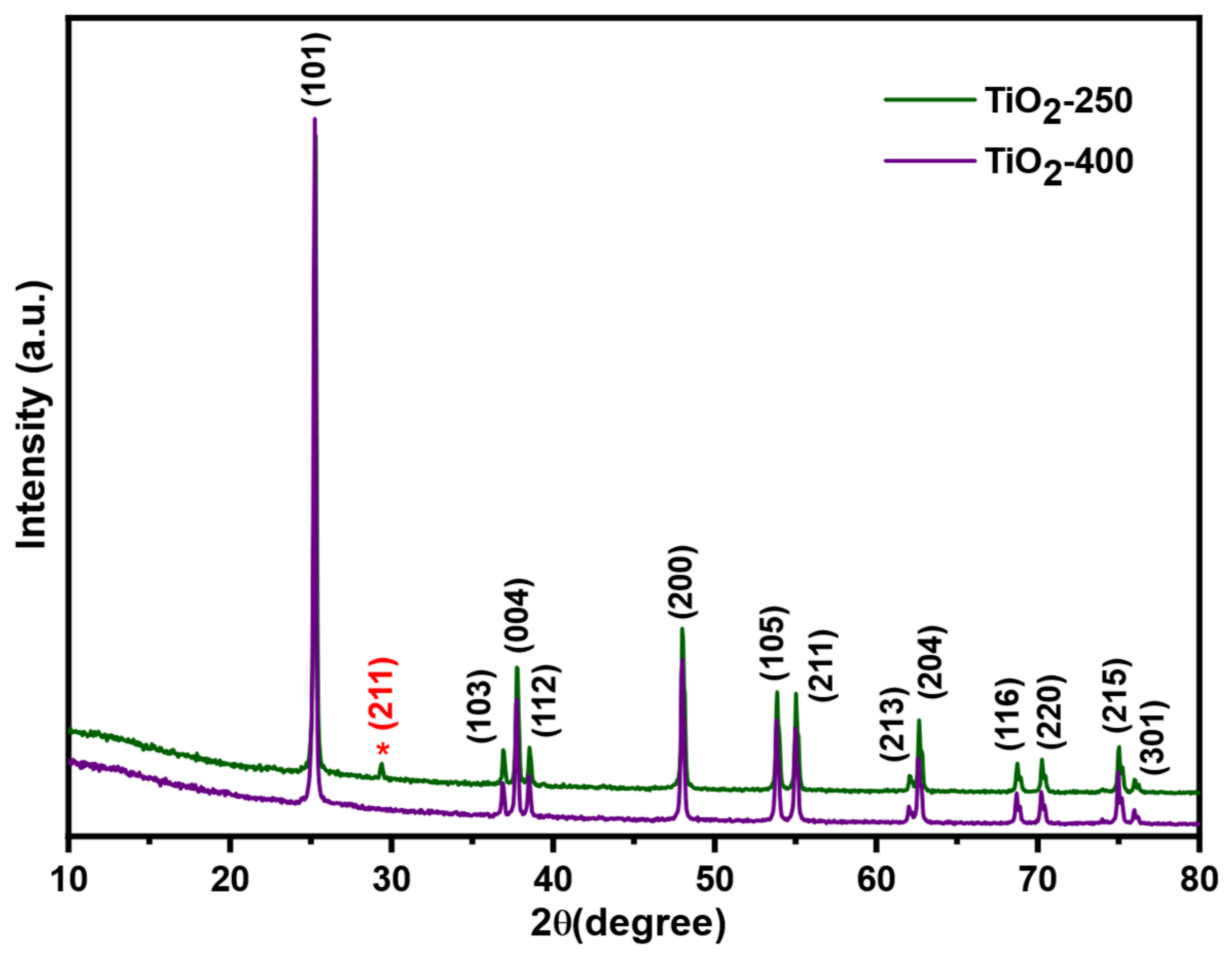
X-ray diffraction patterns of the TiO_2_-250 and TiO_2_-400 nanostructures obtained via sol–gel synthesis. The asterisk (*) indicates the (211) reflection of the brookite phase.

The crystalline structure and phase composition of the synthesized TiO_2_ nanostructures were examined using X-ray diffraction (XRD). Figure 2 shows the diffraction patterns corresponding to the TiO_2_-250 (green curve) and TiO_2_-400 (purple curve) samples for a diffraction angle 2θ ranging from 10° to 80°. Well-defined diffraction peaks characteristic of polycrystalline materials with high crystallinity are observed in both samples. The anatase reflections occur at comparable angular positions in both samples and differ mainly in their relative intensities. The peaks indexed to the lattice planes (101), (103), (004), (112), (200), (105), (211), (213), (204), (116), (220), (215), and (301) match ICDD Standard Card No. 01-071-1166, confirming the predominant formation of the anatase phase with a tetragonal structure and space group *I*4_1_/*amd*.

Meanwhile, the XRD pattern of the TiO_2_-250 sample reveals a low-intensity peak located at 2θ = 29.47°, identified with an asterisk (*) and associated with the (211) plane of the brookite phase according to ICDD card No. 00-002-0514. The presence of this minor phase is consistent with the polymorphic nature of TiO_2_, as the coexistence of anatase and brookite has been frequently reported in materials synthesized via wet processes at low temperatures [18,19]. After post-synthesis heat treatment, this peak is no longer detected, indicating that the brookite contribution falls below the detection limit of the XRD measurement, with anatase remaining as the predominant crystalline phase. Additionally, the heat treatment produced an increase in the intensity of the main peak (101), located at 2θ = 25.20°, accompanied by a narrowing of the diffraction peaks, providing direct evidence of an increase in crystallite size and an improvement in crystalline order [20,21].

Table 1 presents the values obtained using the Harris texture equation (*η_hkl_*) for the quantitative evaluation of the preferential crystallographic orientation of the nanostructures. In both samples, the texture coefficients remain close to unity, indicating a predominantly random distribution of orientations and weak crystallographic texture. The (101) plane exhibits the highest value of *η_hkl_* (1.10 for TiO_2_-250 and 1.07 for TiO_2_-400), indicating a slight preferential orientation associated with the {101} plane family.

After post-synthesis heat treatment, most of the coefficients tend to approach unity. This behavior is particularly evident for the (004), (200), and (204) reflections, whose values change from 1.09, 1.05, and 0.71 to 1.01, 0.99, and 0.91, respectively. These results suggest a more uniform distribution of crystallographic orientations in TiO_2_-400, without the development of an intense texture or any alteration of the slight relative predominance of the (101) reflection.

The dominant presence of the (101) reflection is consistent with the high thermodynamic stability of the (101) surface, which is widely recognized as the anatase facet with the lowest surface energy. This characteristic favors its formation during crystal growth by sol–gel synthesis and explains the relative predominance of this reflection in both samples [22].

**Figure 3:**
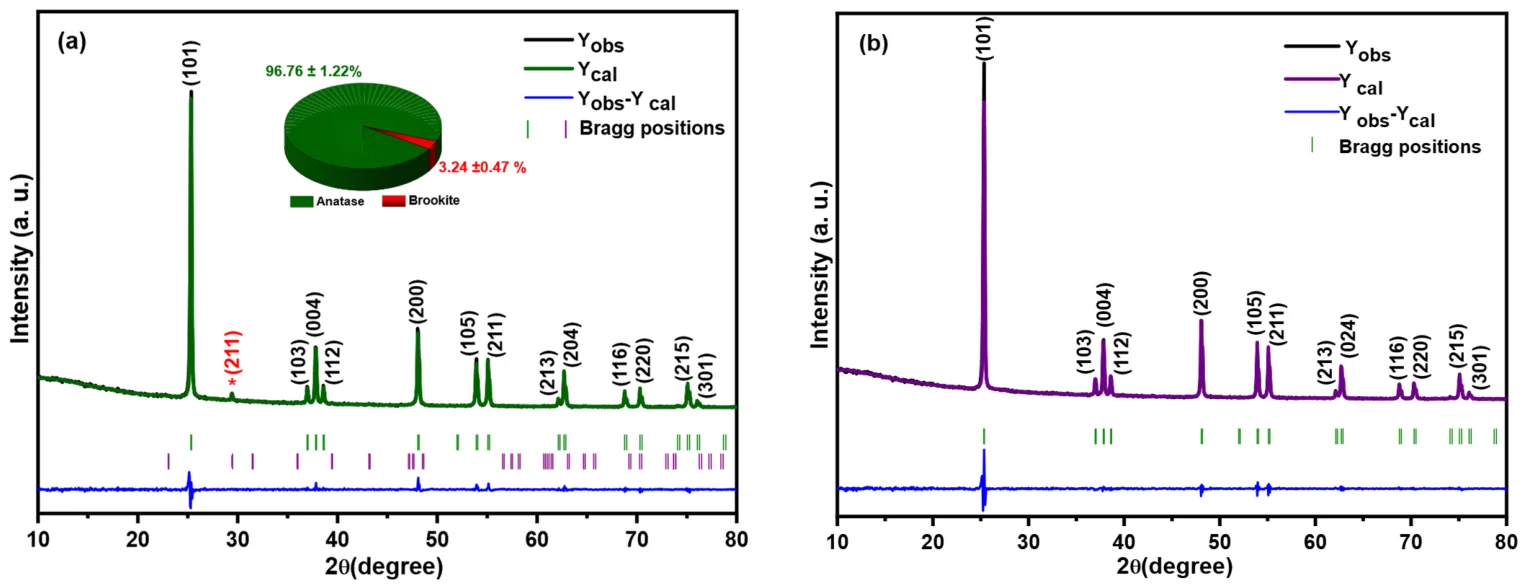
Rietveld refinement of the XRD patterns of (**a**) TiO_2_-250 and (**b**) TiO_2_-400. The observed, calculated and difference profiles are shown together with the Bragg reflection positions of anatase (green, ICDD No. 01-071-1166) and, for TiO_2_-250, brookite (magenta, ICDD No. 00-002-0514). The asterisk (*) indicates the (211) reflection of the brookite phase.

In order to quantify the phase fractions and determine the crystallographic parameters of both samples, the diffraction patterns were refined using the Rietveld method. The refinements obtained for TiO_2_-250 and TiO_2_-400 are presented in Figure 3a,b, respectively. In both cases, the calculated pattern satisfactorily reproduces the experimental pattern throughout the entire angular range analyzed, while the difference curve remains close to zero, demonstrating the good agreement between the crystallographic model and the experimental data. Furthermore, the Bragg positions confirm the correct indexing of the reflections corresponding to the anatase phase and, for the TiO_2_-250 sample, the presence of a minor contribution associated with the brookite phase.

Rietveld refinement made it possible to quantify the phase fractions and determine the crystallographic parameters of both samples (see Table 2). The TiO_2_-250 sample consists of 96.76 ± 1.22 wt.% anatase and 3.24 ± 0.47 wt.% brookite. For TiO_2_-400, no crystalline phase other than anatase was detected within the XRD detection limit. Furthermore, the low values of *R_p_* (<5.1%), *R_wp_* (<6.8%), and χ^2^ (<3.5) demonstrate good agreement between the experimental and calculated diffraction patterns, supporting the reliability of the refined structural parameters.

The minor brookite fraction should, however, be interpreted cautiously. Although the two-phase whole-pattern Rietveld refinement yielded 3.24 ± 0.47 wt.% brookite, this contribution has a relative uncertainty of approximately 14.5% and a Bragg R-factor of 38.4, substantially higher than that of anatase (1.24), indicating that the minor phase is weakly constrained by the diffraction data and should be regarded as a near-detection-limit estimate. In TiO_2_-400, no reflection attributable to brookite was identifiable; therefore, an anatase-only model was used. Accordingly, the results indicate that any residual brookite, if present, is below the XRD detection limit rather than demonstrating a strictly zero brookite fraction.

The refined anatase lattice parameters (Table 2) exhibit a small decrease after post-synthesis heat treatment. The lattice constants changed from a = b = 3.78382 ± 0.00010 Å and c = 9.51122 ± 0.00026 Å for TiO_2_-250 to a = b = 3.78329 ± 0.00007 Å and c = 9.51018 ± 0.00018 Å for TiO_2_-400. Accordingly, the refined unit-cell volume decreased from 136.175 ± 0.006 to 136.122 ± 0.004 Å^3^, corresponding to an absolute variation of 0.053 Å^3^ and a relative decrease of approximately 0.039%. Although this difference is resolved relative to the estimated standard uncertainties obtained from the refinements, its absolute magnitude is small. Therefore, it is conservatively described as a subtle contraction of the refined anatase lattice after heat treatment, without assigning it to a specific microscopic mechanism. The *I*4_1_/*amd* space group remained unchanged, confirming preservation of the tetragonal anatase structure.

**Figure 4:**
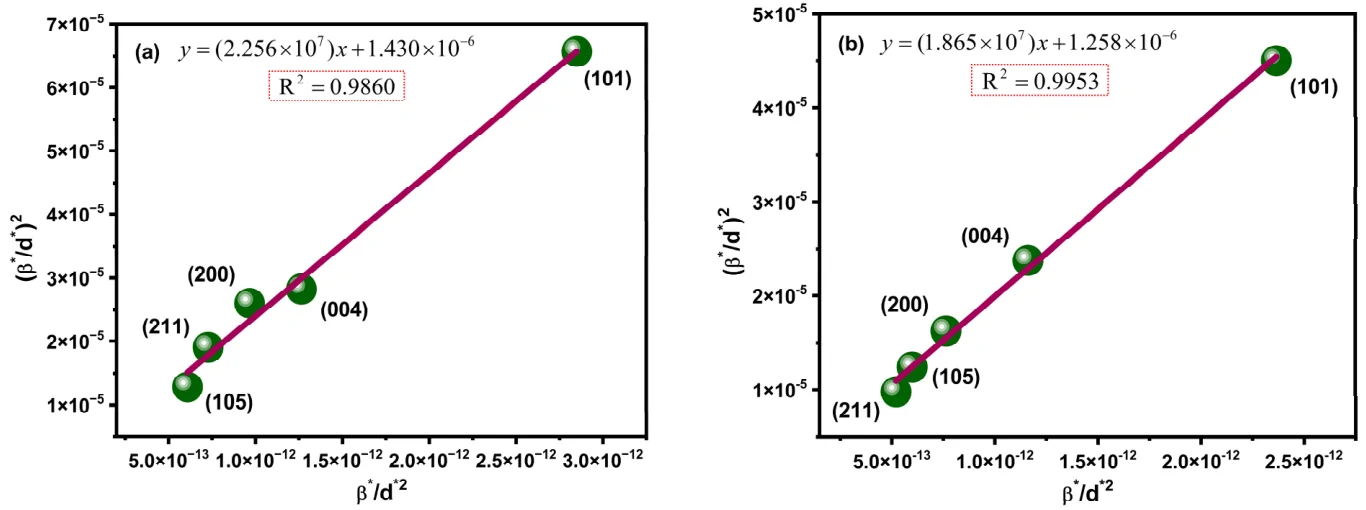
Halder–Wagner analysis of (**a**) TiO_2_-250 and (**b**) TiO_2_-400. Solid lines represent the linear regression fits used to estimate the crystallite size and lattice microstrain.

Figure 4 shows the Halder–Wagner plots for TiO_2_-250 and TiO_2_-400. In both cases, a well-defined linear relationship is observed, with adjusted coefficients of determination of 0.986 and 0.995, respectively, indicating that the broadening of the diffraction peaks can be adequately described by the combined contribution of crystallite size and lattice microstrain.

Post-synthesis heat treatment increased the average crystallite size from 44.3 ± 2.6 nm for TiO_2_-250 to 53.6 ± 1.9 nm for TiO_2_-400, corresponding to an approximate increase of 20.98%. This behavior indicates the growth of coherent crystalline domains during heat treatment and is consistent with the narrowing of the diffraction peaks previously observed in the XRD patterns.

On the other hand, the mean value of the lattice microstrain decreased slightly from 2.4 × 10^−3^ to 2.2 × 10^−3^. This change occurs concurrently with the small contraction of the refined anatase unit cell and may reflect subtle lattice relaxation during heat treatment [23]; however, the present diffraction data do not allow its microscopic origin to be uniquely established.

**Figure 5:**
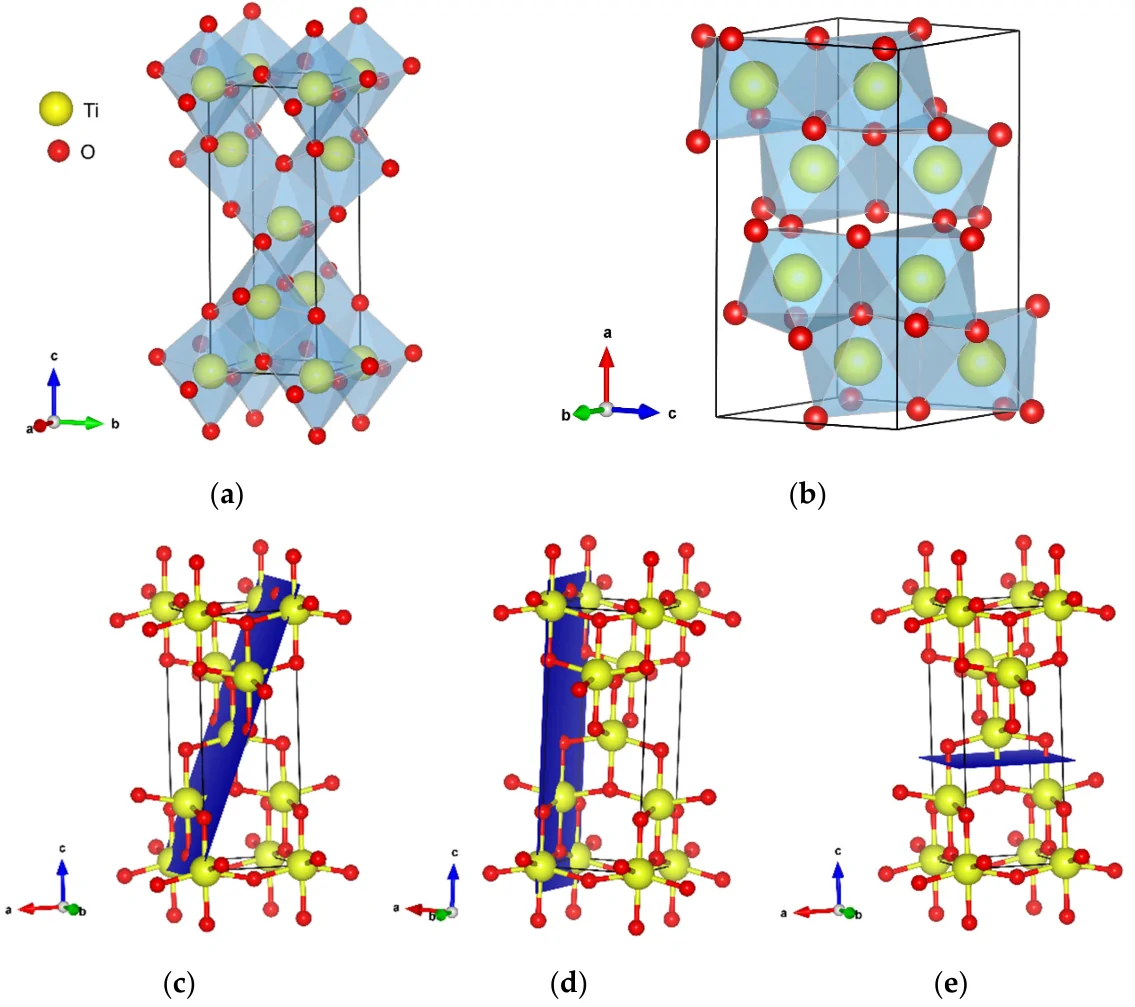
Crystallographic models generated using VESTA: (**a**) anatase unit cell (*I*4_1_/*amd*), (**b**) brookite unit cell (*Pbca*), and crystallographic planes of anatase: (**c**) (101), (**d**) (200), and (**e**) (004).

Figure 5a,b compare the three-dimensional crystal models of the unit cells of anatase and brookite obtained from the refined structural parameters. Anatase crystallizes in the tetragonal space group *I*4_1_/*amd*, while brookite adopts an orthorhombic structure with the space group *Pbca*. In both polymorphs, Ti atoms are octahedrally coordinated by oxygen atoms to form TiO_6_ octahedra; however, the different connectivity of these octahedra gives rise to their distinct crystallographic symmetries and atomic arrangements.

Figure 5c–e illustrate the (101), (200), and (004) crystallographic planes selected based on the texture coefficient analysis. Among these, the (101) plane exhibited the highest diffraction intensity and the highest texture coefficient for both TiO_2_-250 and TiO_2_-400, confirming its dominant contribution to the diffraction patterns. Visualizing these planes provides a direct crystallographic interpretation of the preferred orientation identified by XRD and complements the structural information obtained from Rietveld refinement.

### 3.2. Raman Spectroscopy Analysis

**Figure 6:**
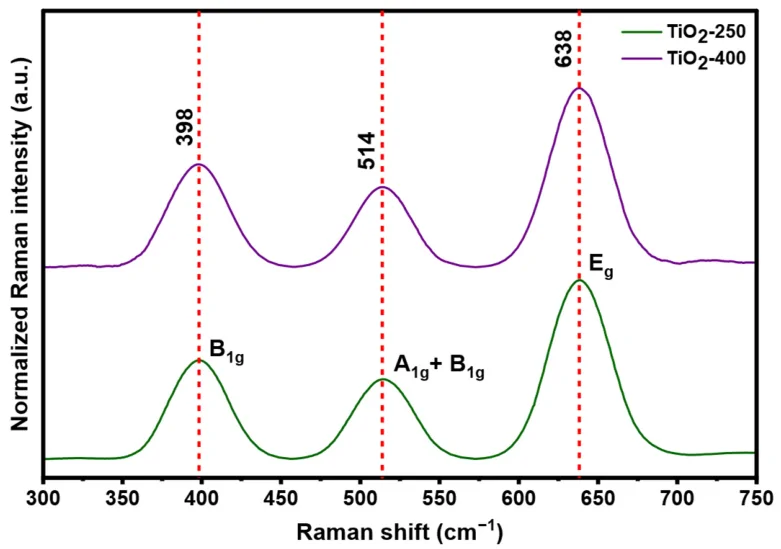
Normalized Raman spectra of TiO_2_-250 and TiO_2_-400. Dashed vertical lines indicate the characteristic *B*_1*g*_, *A*_1*g*_ + *B*_1*g*_, and *E_g_* Raman bands of anatase TiO_2_ located at approximately 398, 514, and 638 cm^−1^, respectively.

Figure 6 shows the Raman spectra of TiO_2_-250 and TiO_2_-400 in the range of 300–750 cm^−1^. Both samples exhibit three well-defined Raman bands, centered at approximately 398, 514, and 638 cm^−1^, characteristic of the anatase phase of TiO_2_. For tetragonal anatase with space group *I*4_1_/*amd*, factorial group analysis predicts six active first-order Raman modes: 3*E_g_* + 2*B*_1*g*_ + *A*_1*g*_. The bands observed in the investigated spectral window are attributed to the *B*_1*g*_ mode near 399 cm^−1^, the unresolved contribution of *A*_1*g*_ + *B*_1*g*_ in the 507–519 cm^−1^ region, and the high-frequency *E_g_* mode near 639 cm^−1^, in good agreement with the characteristic vibrational response reported for the anatase phase of TiO_2_. Ohsaka et al. demonstrated that the Raman band observed around 516 cm^−1^ at room temperature originates from closely spaced contributions, *A*_1*g*_ and *B*_1*g*_, which can be resolved into individual components at lower temperatures, supporting the assignment of the experimentally observed band near 514 cm^−1^ as an unresolved contribution from *A*_1*g*_ + *B*_1*g*_ [24].

The Raman bands originate from first-order optical phonons associated with collective atomic displacements within the TiO_6_ octahedral structure of anatase. In particular, the band near 398 cm^−1^ is attributed to the *B*_1*g*_ symmetry mode, which primarily involves deformation of the O–Ti–O lattice, while the feature near 514 cm^−1^ arises from unresolved contributions from *A*_1*g*_ and *B*_1*g*_ associated with internal distortions of the TiO_6_ octahedra. The high-frequency *E_g_* mode, near 638 cm^−1^, reflects the collective motions of the oxygen sublattice that are strongly coupled to the Ti–O bond lattice. Because Raman frequencies are governed by the local force constants and the symmetry of these collective lattice vibrations, significant changes in the Ti–O bond geometry, octahedral distortion, lattice deformation, or local coordination would generally be expected to produce measurable shifts in the band position and/or line width [25,26,27].

However, according to the parameters reported in Table 3, the experimentally observed differences in Raman shift between TiO_2_-250 and TiO_2_-400 were only 0.60, 0.36, and 0.13 cm^−1^ for the *B*_1*g*_, *A*_1*g*_ + *B*_1*g*_, and *E_g_* bands, respectively, all of which are well below the nominal spectral resolution (4 cm^−1^). Furthermore, the FWHM values showed no systematic narrowing or broadening following heat treatment. These observations suggest that no appreciable changes in the local vibrational response of the anatase lattice are resolved within the instrumental resolution of the present measurements.

This behavior is consistent with the microstructural evolution revealed by XRD. Although the crystallite size increased from 44.3 ± 2.6 to 53.6 ± 1.9 nm and the lattice microstrain decreased slightly, the Raman spectra do not show appreciable changes in the characteristic anatase vibrational response at the available resolution. Therefore, the Raman results are consistent with preservation of the characteristic anatase vibrational signature after heat treatment, while smaller spectral variations below the instrumental resolution cannot be excluded.

### 3.3. X-Ray Photoelectron Spectroscopy (XPS) Analysis

**Figure 7:**
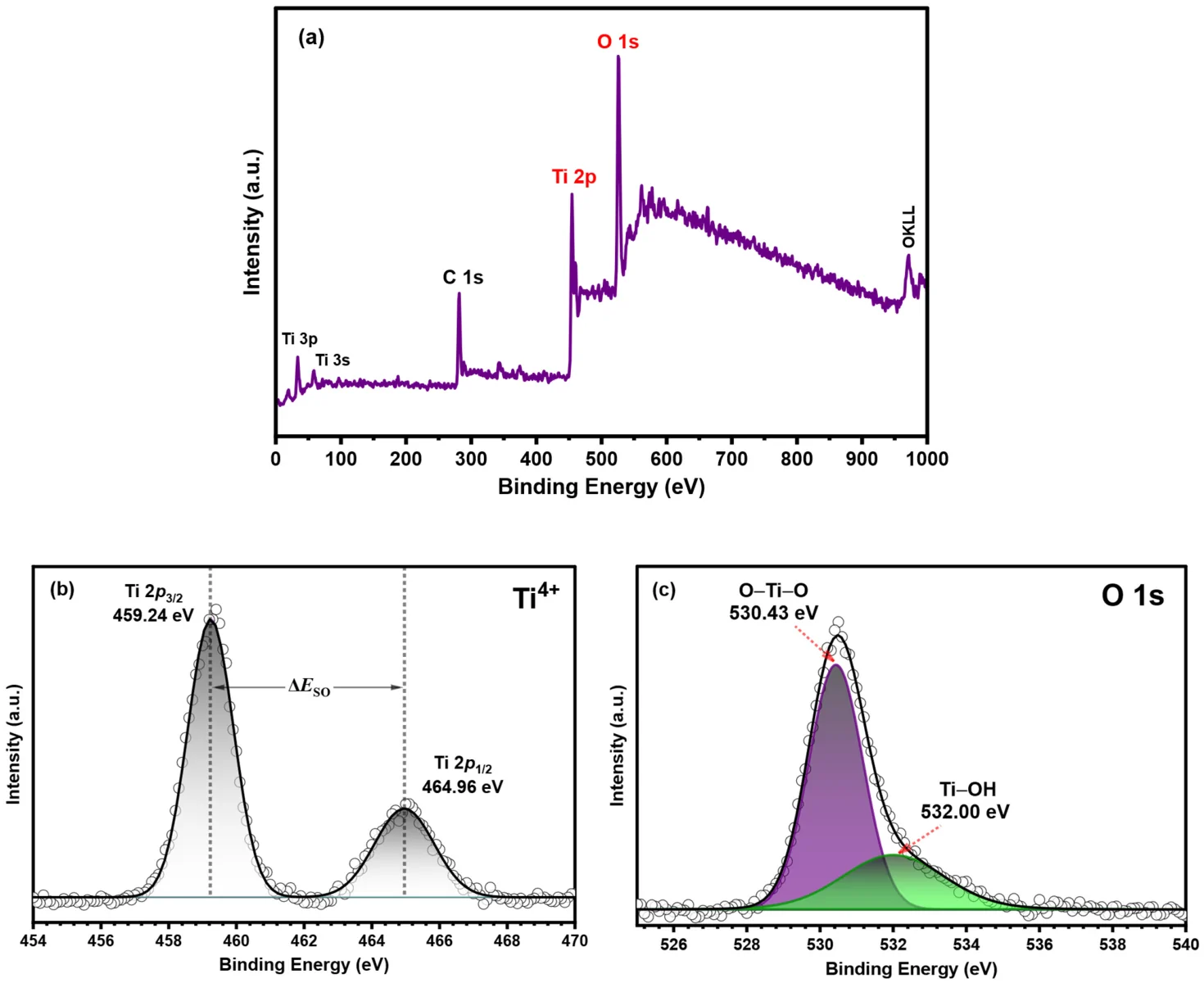
X-ray photoelectron spectroscopy (XPS) analysis of the TiO_2_-400 sample: (**a**) survey spectrum, (**b**) high-resolution Ti 2p spectrum; and (**c**) high-resolution O 1s spectrum.

To determine the surface chemical state of the final heat-treated material, high-resolution X-ray photoelectron spectroscopy (XPS) analysis was performed exclusively on the TiO_2_-400 sample. Structural evolution had been independently evaluated by X-ray diffraction, Rietveld refinement, Halder–Wagner analysis, and Raman spectroscopy, with anatase being the only crystalline phase detected in TiO_2_-400 within the XRD detection limit. Accordingly, XPS was used to characterize the chemical environment of Ti and O on the surface of TiO_2_-400 rather than to establish heat-treatment-induced changes in surface chemistry. Figure 7 shows the survey spectrum together with the high-resolution Ti 2p and O 1s spectra.

The survey spectrum (Figure 7a) reveals the presence of Ti and O as the predominant elements on the surface through the characteristic photoelectron and Auger transitions of Ti 3p, Ti 3s, Ti 2p, O 1s, and O KLL, respectively. A C 1s signal was also detected, which is typically associated with adventitious carbon adsorbed during atmospheric exposure of the sample. No additional photoelectron peaks attributable to other chemical elements were detected within the instrument’s sensitivity range, indicating the absence of detectable surface contaminants introduced during synthesis or post-synthesis heat treatment. Therefore, the survey spectrum establishes the elemental composition of the TiO_2_-400 surface and provides a suitable basis for high-resolution analysis of the Ti 2p and O 1s core levels.

The high-resolution Ti 2p spectrum (Figure 7b) shows the spin–orbit doublet characteristic of titanium, arising from the splitting of the inner Ti 2p electronic level into the Ti 2p_3_/_2_ and Ti 2p_1_/_2_ components, located at 459.24 and 464.96 eV, respectively. The spin–orbit splitting (ΔE_so_ = 5.72 eV) is fully consistent with the characteristic electronic structure of Ti^4+^ in TiO_2_ described in the literature, supporting the assignment of titanium on the TiO_2_-400 surface predominantly to the Ti^4+^ oxidation state characteristic of anatase TiO_2_ [28]. The fitting parameters obtained for both components are summarized in Table 4.

The well-defined and symmetrical line shapes of both components, together with the absence of additional low-binding-energy contributions attributable to reduced titanium (Ti^3+^) species, indicate that titanium is predominantly present in its fully oxidized state within the detection limit of the XPS technique [28,29]. These results indicate that the TiO_2_-400 surface is dominated by Ti^4+^ species, consistent with the Ti–O network characteristic of anatase TiO_2_.

The high-resolution O 1s spectrum (Figure 7c) was deconvoluted into two chemically distinct components, centered at 530.43 ± 0.01 and 532.00 ± 0.37 eV, whose fitting parameters are summarized in Table 4. The component with the lower binding energy is assigned to lattice oxygen (Ti–O–Ti), corresponding to O^2–^ ions incorporated into the TiO_2_ crystal lattice, while the component with the higher binding energy is attributed predominantly to surface hydroxyl species (–OH). The 1.57 eV separation between the two components is consistent with the ranges reported for lattice oxygen and hydroxyl groups on TiO_2_ surfaces [30].

Analysis of the integrated areas reveals that the lattice oxygen accounts for 69.52% of the total O 1s signal, while surface hydroxyl species contribute 30.48%, indicating that the surface chemistry is primarily governed by the Ti–O–Ti network, although it retains a significant fraction of hydroxyl groups associated with the high surface reactivity of nanostructured TiO_2_. The presence of these surface species after heat treatment is consistent with the adsorption and partial dissociation of water on anatase surfaces exposed to the environment; therefore, it does not necessarily imply that hydroxyl groups persisted during heating [31]. Likewise, the component at 532.00 eV is not assigned to oxygen vacancies, since high-binding-energy O 1s signals do not, by themselves, constitute direct evidence or allow for the quantification of such defects [32].

Taken together, the XPS results characterize the surface of TiO_2_-400 as being dominated by Ti^4+^ species and Ti–O–Ti lattice oxygen, together with a contribution from surface hydroxyl species associated with interaction with the environment. No additional low-binding-energy Ti 2p component attributable to Ti^3+^ was resolved within the detection capability of the measurement. Because XPS was performed only on TiO_2_-400, these data do not permit a direct comparison of surface chemistry before and after heat treatment and therefore are not used to infer heat-treatment-induced changes in the Ti oxidation state or surface reduction. Instead, the XPS results provide chemical-state characterization of the final heat-treated material, complementary to the long-range structural information from XRD and the local vibrational information from Raman spectroscopy.

### 3.4. FE-SEM, EDS, and Morphometric Analysis

**Figure 8:**
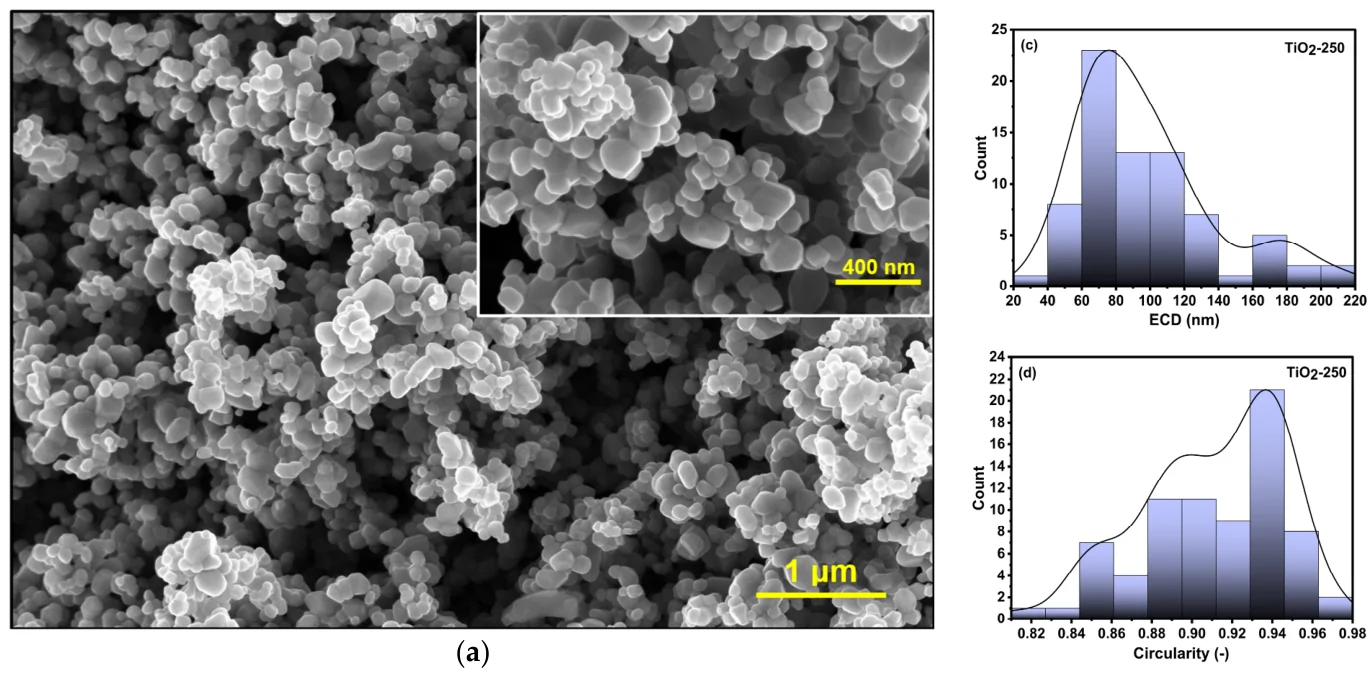

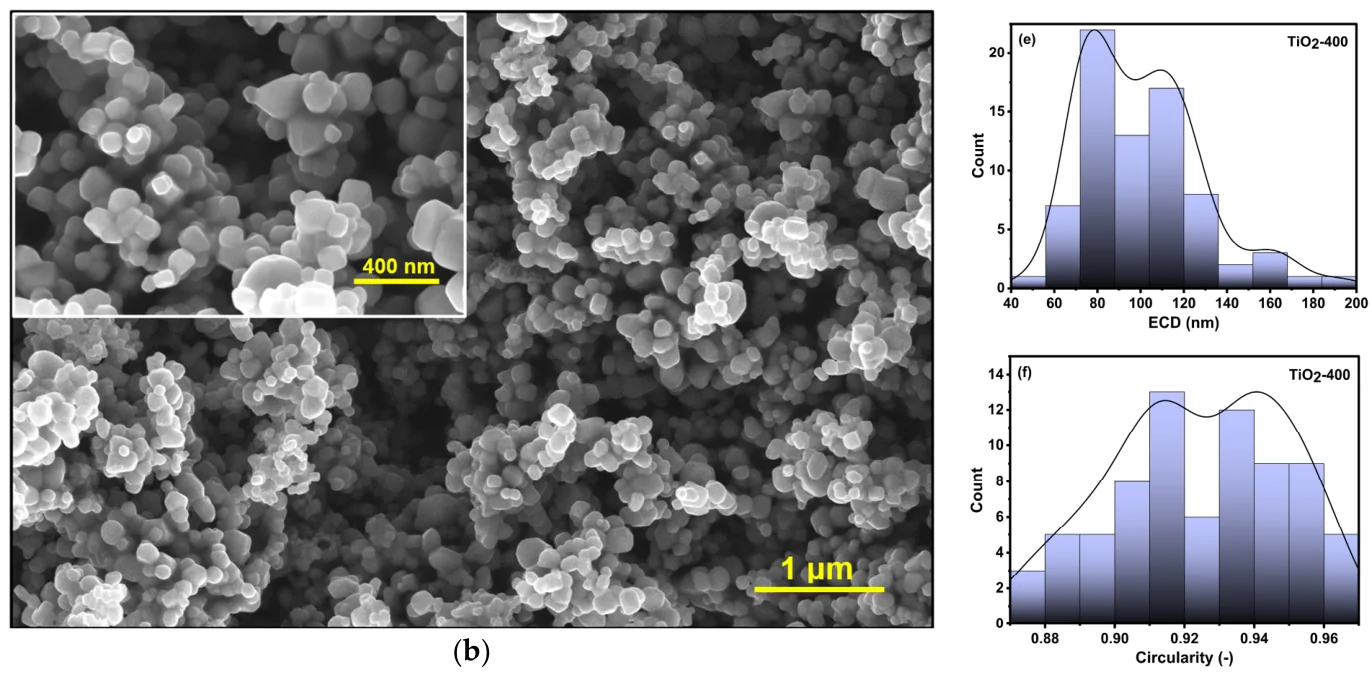
FE-SEM micrographs and quantitative morphological analysis of the TiO_2_ nanostructures: (**a**) TiO_2_-250 and (**b**) TiO_2_-400. The main micrographs were acquired at 30,000×, while the insets show representative images acquired at 100,000×. The corresponding histograms (**c**–**f**) show the equivalent circular diameter (ECD) and circularity distributions obtained from the analysis of 75 particles per sample; the solid lines represent kernel density estimates obtained using Scott’s bandwidth selection criterion.

FE-SEM micrographs of TiO_2_-250 and TiO_2_-400 reveal that both samples exhibit a morphology consisting of strongly agglomerated nanoscale particles, organized into irregular aggregates with interparticle spaces distributed throughout the structure (Figure 8). In TiO_2_-250, a heterogeneous population of predominantly equiaxial particles is observed, with rounded to slightly faceted contours and a noticeable degree of agglomeration. Higher-magnification images allow for a clearer distinction of the individual particles that make up these aggregates, revealing variations in their dimensions and geometry. After heat treatment up to 400 °C, TiO_2_-400 retains the agglomerated architecture and general morphology observed in TiO_2_-250, with no evidence of major morphological transformations or the formation of distinct anisotropic structures. However, the TiO_2_-400 particles visually exhibit slightly more regular contours and an apparently more uniform distribution, suggesting subtle morphological changes after heat treatment without substantial alteration of the overall particle architecture.

The quantitative morphometric analysis performed on 75 particles per sample allowed for a more precise evaluation of the changes induced by heat treatment. The equivalent circular diameter (ECD) had mean values of 97.18 ± 40.22 nm for TiO_2_-250 and 100.63 ± 28.01 nm for TiO_2_-400, while the medians were 84.42 and 96.03 nm, respectively. Although the mean value increased slightly after heat treatment, the comparison using the nonparametric Mann–Whitney test did not reveal any statistically significant differences between the two size distributions (*p* = 0.0982). In contrast, circularity increased from 0.910 ± 0.035 for TiO_2_-250 to 0.924 ± 0.025 for TiO_2_-400, with medians of 0.913 and 0.923, respectively, and this difference was statistically significant (*p* = 0.0221). These results indicate that the heat treatment did not produce a statistically detectable change in particle size but was accompanied by a slight, yet significant, shift toward more regular projected contours. The morphometric parameters obtained for both samples, along with the results of the statistical comparison, are summarized in Table 5.

As shown in Table 5, the analysis of complementary geometric descriptors confirmed that the overall morphology of the particles remained essentially stable after heat treatment. The aspect ratio (AR) was 1.173 ± 0.140 for TiO_2_-250 and 1.168 ± 0.103 for TiO_2_-400, with no statistically significant differences between the two distributions (*p* = 0.4786). These values, which are relatively close to unity, are consistent with the predominantly equiaxial nature observed by FE-SEM and show that the heat treatment did not induce any appreciable elongation of the particles. Similarly, the solidity remained virtually unchanged, with values of 0.9910 ± 0.0047 and 0.9916 ± 0.0046 for TiO_2_-250 and TiO_2_-400, respectively (*p* = 0.2119). The high solidity values, close to unity, indicate highly compact projected contours with few concavities. In this regard, the absence of statistically significant differences in ECD, AR, and solidity, together with the significant increase in circularity, indicates that heat treatment up to 400 °C essentially preserved the size, elongation, and compactness of the particles, producing only a subtle shift toward more regular projected contours.

From a mechanistic perspective, the results obtained are consistent with an evolution dominated by thermally induced intraparticle crystalline reorganization, rather than by pronounced external particle growth. The initial coexistence of anatase and a minor fraction of brookite in TiO_2_-250 can be understood based on the close crystallographic relationship between the two polymorphs, whose structures consist of TiO_6_ octahedra that differ in their arrangement. Some studies have demonstrated the existence of anatase–brookite interfaces and intergrowths in nanocrystalline TiO_2_, showing that the interconversion between the two structures may involve local displacements of Ti cations toward adjacent octahedral sites, accompanied by the propagation of the crystallographic interface between the two polymorphs [33]. In the present study, heat treatment up to 400 °C was accompanied by the loss of the detectable brookite contribution in the XRD pattern, with anatase remaining as the only crystalline phase identified within the XRD detection limit. This observation is consistent with a reduction in the brookite fraction below the XRD detection threshold, but does not by itself demonstrate complete transformation of brookite.

A thermal transformation from brookite to anatase has been observed experimentally in TiO_2_ nanoparticles subjected to calcination treatments, accompanied by an increase in crystallinity and in the size of the crystalline domains [34]. In this case, this progressive selection of anatase occurs simultaneously with the growth of coherently diffracting domains, whose size increases from 44.3 ± 2.6 to 53.6 ± 1.9 nm, while the ECD remains statistically invariant. This decoupled response between the crystallographic scale and the external morphological scale indicates that the predominant effect of the heat treatment took place within the particles, promoting the growth and reorganization of the anatase domains without requiring a proportional expansion of their outer envelope.

The initial formation of anatase/brookite mixtures and their subsequent evolution depend heavily on the nucleation, growth, and aggregation conditions imposed during the sol–gel synthesis proposed in this study, which explains the sensitivity of these polymorphs to the chemical and thermal conditions of the system [35]. In this regard, the loss of the detectable brookite contribution, the increase in the size of the crystalline domains, and the dimensional stability observed by FE-SEM support an evolution toward a structurally more homogeneous and crystallographically more developed anatase, induced by the post-synthesis heat treatment.

**Figure 9:**
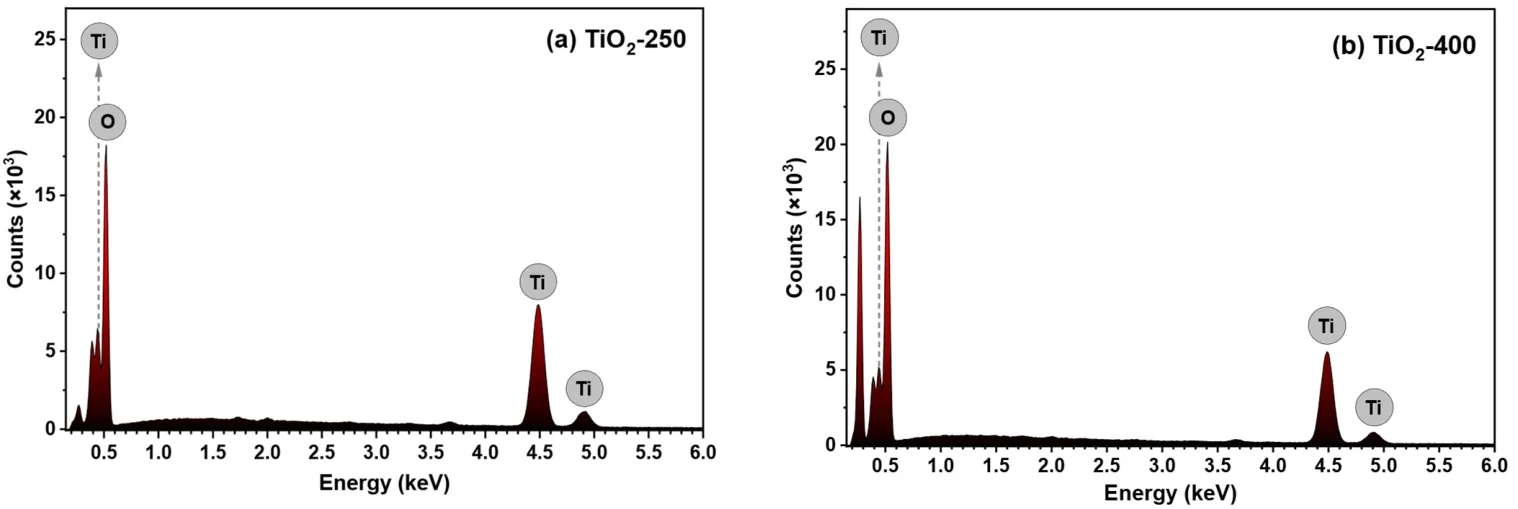
EDS spectra of the TiO_2_ nanostructures: (**a**) TiO_2_-250 and (**b**) TiO_2_-400.

Complementary to the morphological analysis, the elemental composition of the nanostructures was evaluated using EDS. As shown in Figure 9a,b, the spectra corresponding to TiO_2_-250 and TiO_2_-400 exhibit the characteristic emissions of O and Ti, with the O Kα signal in the low-energy region and the Ti Kα and Ti Kβ lines located at approximately 4.5 and 4.9 keV, respectively. The predominance of these signals in both samples confirms Ti and O as the main detected elements and provides compositional evidence complementary to the structural identification of TiO_2_ by XRD.

The elemental analysis presented in Table 6 shows O and Ti contents of 57.99 ± 0.58 at.% and 42.01 ± 0.22 at.% for TiO_2_-250, while TiO_2_-400 has 68.31 ± 0.40 at.% O and 31.69 ± 0.18 at.% Ti, corresponding to O/Ti atomic ratios of approximately 1.38 and 2.16, respectively. These values are reported as semiquantitative descriptors obtained under identical EDS acquisition and normalization conditions and are not interpreted as direct measurements of bulk stoichiometry.

Because the EDS quantification was normalized exclusively to Ti and O, the apparent decrease in the Ti atomic percentage from 42.01 to 31.69 at.% does not represent a physical loss of titanium; it reflects the corresponding change in the normalized relative contribution of oxygen. Moreover, the quantification of O by EDS is subject to substantial uncertainty associated with low-energy X-ray emission, absorption effects, and matrix corrections [36,37]. Consequently, the O/Ti ratios are not used to infer a more complete Ti–O lattice, absolute stoichiometry, or changes in oxygen-vacancy concentration.

The C 1s signal detected by the surface-sensitive XPS measurement of TiO_2_-400 was assigned to adventitious carbon; however, carbon was not included in the EDS quantitative protocol. EDS samples a larger interaction volume than XPS, and XPS data were not acquired for TiO_2_-250. Therefore, the EDS and XPS datasets are not combined to derive comparative stoichiometric O/Ti ratios between the two samples. In the present work, EDS is used primarily to confirm the predominance of Ti and O in both samples and to provide a semiquantitative comparison of Ti and O under identical acquisition conditions.

### 3.5. UV–Vis Diffuse Reflectance and Optical Analysis

**Figure 10:**
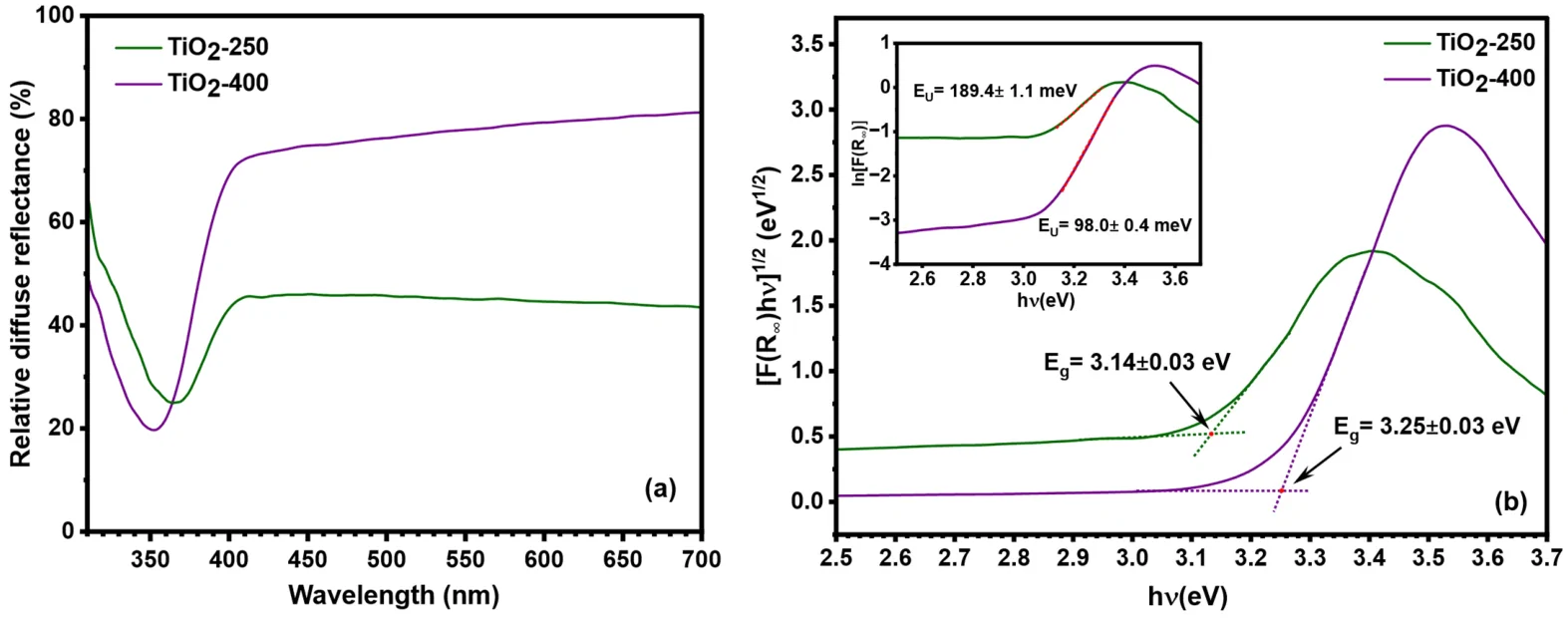
Optical response of TiO_2_-250 and TiO_2_-400: (**a**) relative diffuse reflectance spectra; (**b**) Tauc plots obtained from the Kubelka–Munk function assuming an indirect allowed transition. The band gap energy was determined from the intersection between the linear pre-edge baseline and the linear Tauc region. The inset in panel (**b**) shows the Urbach analysis used to determine the Urbach energy.

The relative diffuse reflectance spectra of TiO_2_-250 and TiO_2_-400 show a distinct change in optical response following heat treatment (Figure 10a). TiO_2_-250 exhibits a response shifted toward longer wavelengths and lower relative reflectance in the visible region, while TiO_2_-400 exhibits a shift of the optical edge toward shorter wavelengths.

Because the diffuse reflectance of particulate materials depends on both absorption and scattering, the absolute differences in reflectance cannot be interpreted solely as changes in the absorption coefficient; however, the observed shift in the optical edge between the two samples confirms a thermally induced change in the optical response.

The experimental reflectance data were transformed using the Kubelka–Munk function and analyzed using the Tauc formalism for an allowed indirect transition. To account for the pre-edge contribution observed in both samples, *E_g_* was determined from the intersection of the linear fit of the pre-edge region and the linear fit corresponding to the Tauc edge (Figure 10b). This procedure yielded apparent optical band gap values of 3.14 ± 0.03 eV for TiO_2_-250 and 3.25 ± 0.03 eV for TiO_2_-400, corresponding to an increase of approximately 0.11 eV following heat treatment. The greater pre-edge contribution observed for TiO_2_-250, together with its higher Urbach energy, is consistent with a broader exponential absorption tail. The corresponding microscopic origin, including the possible contribution of localized electronic states, cannot be uniquely established from the present optical measurements.

In addition, the Urbach analysis shown in the inset of Figure 10b revealed a decrease in *E_U_* from 189.4 ± 1.1 meV to 98.0 ± 0.4 meV, equivalent to a reduction of approximately 48%. The final value of *E_g_* closely approximates the widely reported value of ~3.2 eV for anatase, whereas multiphase TiO_2_ systems and materials containing brookite may exhibit optical edges shifted toward lower energies. For example, Leal et al. reported values close to 3.0 eV for brookite and ~3.2 eV for anatase [38], whereas Giampiccolo et al. obtained an indirect band gap of 2.95 eV for a sol–gel TiO_2_ composed of 64.1 wt.% anatase and 35.9 wt.% brookite [39].

The increase in *E_g_* can be interpreted in terms of changes in the material’s optical edge. In TiO_2_, the valence band is dominated primarily by O 2p states and the conduction band by Ti 3d states; local disorder, defects, and structural heterogeneities can introduce localized states within or near the band gap, extending the absorption to energies below the interband edge. In TiO_2_-250, the coexistence of anatase and brookite, together with the higher *E_U_* value, is consistent with a broader energy distribution of tail states. After heat treatment, the lower *E_U_* and the shift of the corrected Tauc intersection toward higher energy occur concurrently with the loss of the detectable brookite contribution and the increase in crystalline order. This co-evolution is consistent with a narrower distribution of localized states near the optical edge; however, the present data do not establish that polymorphic homogenization or coherent-domain growth is the direct cause of the band-tail narrowing. Previous studies have reported that TiO_2_ with higher defectivity or a multiphase composition can simultaneously exhibit higher Urbach energies and lower apparent optical gaps [8].

From a chemical perspective, studies of TiO_2_ subjected to different atmospheres have shown that reducing conditions, which favor oxygen vacancies and Ti^3+^ species, can broaden absorption into the visible region and decrease the apparent band gap, whereas materials treated under oxidizing conditions may exhibit an optical response closer to that of stoichiometric anatase [40,41]. In the present study, however, XPS was performed only on TiO_2_-400 and therefore cannot establish whether the heat treatment altered the Ti oxidation state or the concentration of oxygen-vacancy-related surface species relative to TiO_2_-250. The Ti^4+^-dominated XPS spectrum consequently characterizes only the final surface state and is not used as evidence for a heat-treatment-induced change in surface chemistry. The experimentally observed decrease in *E_U_* is instead treated as optical evidence of a narrower distribution of band-tail states, while attribution of this change to a specific surface-defect mechanism remains an interpretation that is not directly demonstrated by the available XPS data.

In addition, the optical evolution is closely correlated with the structural change determined by XRD: TiO_2_-250 transitions from 96.76 ± 1.22 wt.% anatase + 3.24 ± 0.47 wt.% brookite to a state in which anatase is the only crystalline phase detected in TiO_2_-400 within the XRD detection limit, while the size of the crystalline domains increases from 44.3 ± 2.6 to 53.6 ± 1.9 nm. However, the increase in *E_g_* is not directly attributed to the growth of crystallites. Quantum confinement effects in TiO_2_ are primarily relevant at dimensions considerably smaller than those observed here, and annealing studies have even reported decreases in *E_g_* as crystal size increases, demonstrating that there is no universal relationship between these two variables [42,43,44]. In this study, domain growth and gap widening are, therefore, concurrent manifestations of thermal reorganization, while the reduction in *E_U_* provides direct optical evidence of a narrower band-tail distribution.

Overall, the key finding is not simply an increase in *E_g_* with temperature. Following heat treatment, TiO_2_-250, which contains anatase and a minor brookite fraction and exhibits a broadened optical edge (*E_U_* = 189.4 meV), evolves to TiO_2_-400, in which anatase is the only crystalline phase detected within the XRD detection limit, while *E_U_* decreases to 98.0 meV and *E_g_* shifts from 3.14 to 3.25 eV. These structural and optical changes occur concurrently without a significant change in the external particle size, supporting a multiscale correlation between polymorphic evolution, intraparticle crystalline reorganization, and optical-edge narrowing rather than a direct causal relationship between polymorphic homogenization and band-tail narrowing.

## 4. Conclusions

Post-synthesis heat treatment was associated with a clear structural evolution of the TiO_2_ nanostructures obtained via the sol–gel method. TiO_2_-250, consisting of 96.76 ± 1.22 wt.% anatase and 3.24 ± 0.47 wt.% brookite, evolved to a state in which anatase was the only crystalline phase detected in TiO_2_-400 within the XRD detection limit, accompanied by an increase in the size of the crystalline domains from 44.3 ± 2.6 to 53.6 ± 1.9 nm. However, the ECD showed no statistically significant changes, indicating that the thermal evolution occurred predominantly through intraparticle crystalline reorganization rather than appreciable external particle growth. Raman spectroscopy showed no appreciable changes in the characteristic anatase vibrational response within the 4 cm^−1^ instrumental resolution, whereas XPS characterized the TiO_2_-400 surface as predominantly Ti^4+^. Because XPS was not acquired for TiO_2_-250, no before-and-after change in surface chemistry is inferred from these measurements.

The structural reorganization was accompanied by a clear modification of the optical response. The apparent optical band gap increased from 3.14 ± 0.03 to 3.25 ± 0.03 eV, while the Urbach energy decreased from 189.4 ± 1.1 to 98.0 ± 0.4 meV. The simultaneous blue shift of the optical edge and decrease in Urbach energy indicate a narrowing of the exponential absorption tail. Although this behavior is commonly associated with changes in electronic disorder and localized tail states, the present measurements do not directly determine their microscopic origin or population. Overall, moderate heat treatment was accompanied by concurrent polymorphic homogenization, coherent-domain growth, and band-tail narrowing without a statistically significant change in the external particle dimensions. These observations establish a multiscale correlation between crystallographic evolution and optical response, but do not by themselves demonstrate that polymorphic homogenization directly causes the narrowing of the Urbach tail.

## Figures and Tables

**Table 1:** Normalized diffraction intensities and texture coefficient (*η_hkl_*) of the main anatase reflections for TiO_2_-250 and TiO_2_-400, compared with the ICDD reference pattern (No. 01-071-1166).

(*hkl*)	Intensity Norm. (%)			*η_hkl_* TiO_2_-250	*η_hkl_* TiO_2_-400
	Exp. TiO_2_-250	Exp. TiO_2_-400	ICDD		
(101)	100	100.0	100.0	1.10	1.07
(004)	19.0	17.5	18.6	1.09	1.01
(200)	23.6	22.5	24.2	1.05	0.99
(105)	14.3	14.7	15.0	1.03	1.05
(211)	13.7	13.6	14.8	1.00	0.98
(204)	7.18	9.3	11.0	0.71	0.91

**Table 2:** Phase composition, refined crystallographic parameters, and reliability factors obtained from the Rietveld refinement of TiO_2_-250 and TiO_2_-400. ND: not detected within the XRD detection limit.

Sample	Anatase (%)	Brookite (%)	a (Å)	b (Å)	c (Å)	V (Å^3^)	*R_p_* (%)	*R_wp_* (%)	*R_exp_* (%)	χ^2^
TiO_2_-250	96.76 ± 1.22	3.24 ± 0.47	3.78382 (10)	3.78382 (10)	9.51122 (26)	136.175 (6)	4.71	6.56	3.66	3.22
TiO_2_-400	100	ND	3.78329 (7)	3.78329 (7)	9.51018 (18)	136.122 (4)	5.00	6.74	3.65	3.41

**Table 3:** Raman band positions and FWHM obtained for TiO_2_-250 and TiO_2_-400.

Raman Mode	Reference (cm^−1^) [24]	TiO_2_-250		TiO_2_-400	
		Raman Shift (cm^−1^)	FWHM (cm^−1^)	Raman Shift (cm^−1^)	FWHM (cm^−1^)
*B*_1*g*_	~399	398.40 ± 0.18	39.83 ± 0.41	397.80 ± 0.14	40.77 ± 0.30
*A*_1*g*_ + *B*_1*g*_	~507–519	514.44 ± 0.21	40.50 ± 0.48	514.08 ± 0.15	38.79 ± 0.34
*E_g_*	~639	638.41 ± 0.10	43.81 ± 0.24	638.28 ± 0.07	42.54 ± 0.14

**Table 4:** Fitting parameters obtained from the deconvolution of the high-resolution Ti 2p and O 1s XPS spectra of the TiO_2_-400 sample.

Core Level	Component	Binding Energy (eV)	FWHM (eV)	Peak Area (%)
Ti 2p	Ti 2p**_3/2_** (Ti**^4+^**)	459.24 ± 0.01	1.59 ± 0.01	70.83
Ti 2p	Ti 2p**_1/2_** (Ti**^4+^**)	464.96 ± 0.02	2.06 ± 0.40	29.17
O 1s	Ti–O–Ti	530.43 ± 0.01	1.74 ± 0.05	69.52
O 1s	–OH	532.00 ± 0.37	3.28 ± 0.48	30.48

**Table 5:** Quantitative morphological parameters of TiO_2_-250 and TiO_2_-400 obtained from FE-SEM image analysis, together with the crystallite size determined by XRD.

Sample	Crystallite Size (nm)	ECD (nm)	Circularity	Aspect Ratio	Solidity
TiO_2_-250	44.3 ± 2.6	97.18 ± 40.22	0.910 ± 0.035	1.173 ± 0.140	0.9910 ± 0.0047
TiO_2_-400	53.6 ± 1.9	100.63 ± 28.01	0.924 ± 0.025	1.168 ± 0.103	0.9916 ± 0.0046
*p*-value	—	0.0982	0.0221 *	0.4786	0.2119

* Statistically significant difference between TiO_2_-250 and TiO_2_-400 (*p* < 0.05).

**Table 6:** Elemental composition of TiO_2_-250 and TiO_2_-400 determined by EDS analysis.

Element	TiO_2_-250		TiO_2_-400	
	Weight %	Atom %	Weight %	Atom %
O	31.56 ± 0.32	57.99 ± 0.58	41.86 ± 0.24	68.31 ± 0.40
Ti	68.44 ± 0.35	42.01 ± 0.22	58.14 ± 0.33	31.69 ± 0.18
Total	100.00	100.00	100.00	100.00

## Data Availability

Data are contained within the article.
